# Usability of Eye-Gaze Controlled Computers in Sweden: A Total Population Survey

**DOI:** 10.3390/ijerph17051639

**Published:** 2020-03-03

**Authors:** Helena Hemmingsson, Maria Borgestig

**Affiliations:** 1 Department of Special Education, Stockholm University, Se-106 91 Stockholm, Sweden; 2 Department of Health, Medicine and Caring Sciences, Linköping University, 581 83 Linköping, Sweden; 3 Faculty of Medicine and Health, School of Health Sciences, Örebro University, 702 81 Örebro, Sweden; Maria.Borgestig@oru.se

**Keywords:** adults, children, complex communication needs, eye-gaze control devices, total population survey

## Abstract

Eye-gaze technology allows individuals with severe physical disabilities and complex communication needs to control a computer or other devices with eye-gaze, thereby enabling them to communicate and participate in society. To date, most research on eye-gaze controlled devices related to persons with disabilities has focused on a single diagnosis in either adults or children and has included only a few participants. This current study utilized a total population survey to identify the prevalence and perceived usability of eye-gaze technology among adults and children in Sweden. Participants were 171 eye-gaze technology users with severe physical and communication impairments, ranging between 4 and 81 years. Cerebral palsy was the most common diagnosis. Daily usage was found in 63%, while 33% had weekly, and 4% had less frequent usage. Adults, compared with children, reported using their computers more frequently (65%/38%; *p* < 0.01), and for the activities they needed to perform (59%/31%; *p* < 0.01) and were more satisfied with services, indicating that service providers should prioritize and develop more effective services for children and their parents.

## 1. Introduction

Eye-gaze technology provides individuals who have severe physical disabilities and complex communication needs with opportunities to participate in both the digital and the social world. Users are able to interact with objects on a computer screen simply by moving their eyes rather than using a mouse or keyboard. This is made possible by a camera mounted on the screen that reads, within a few millimeters, where on the screen the person is gazing [1,2]. 

Although computers controlled with eye-gaze have been discussed as an assistive technology since the 1990s, it has only been since the early 2000s that eye-gaze controlled devices reliable enough to be used outside laboratory settings have become available [3]. Access has been limited due to the high cost of the technology [4] and lack of research on usability for people with disabilities, which may have raised concerns of device abandonment when the novelty of eye-gaze controlled devices subsides. As a result, little is known about who uses the eye-gaze controlled computer, including its application and perceived usability in everyday life [4,5,6]. 

The concept of usability is often used in assistive technology research and refers to perceptions about how well the design of an environment or product enables functioning, performance, and well-being; it is a measure of the degree of effectiveness, efficiency, and user satisfaction [7,8]. In the current study, usability is based on the definition of the International Organization for Standardization [9], which defines it as “the extent to which a system, product, or service can be used by specified users to achieve specified goals with effectiveness, efficiency, and satisfaction in a specified context of use.” Additionally, assistive technology usage (e.g., duration, frequency of use) is regarded as a quality aspect of usability, which is recommended by scholars in the field [10,11].

Mele and Federici [6] showed that research into technical aspects of eye-gaze control is comprehensive but limited with regard to people with disabilities. A recent literature review [5], overall, found two interventions studies examining the effectiveness of the eye-gaze controlled computer for facilitating communication for people with significant physical disabilities (Hwang et al. [12] and Borgestig et al. [13]). With few exceptions, studies on eye-gaze controlled computer for people with disabilities are either cross-sectional or case studies that include only a few participants. Early research explored the potential of the eye-gaze controlled computer for different groups of people with disabilities and described contextual circumstances, adjustment, and software problems, as well as positioning and seating considerations for optimal success [14,15,16]. More recent research has largely focused on communication skills and requirements of adults with amyotrophic lateral sclerosis (ALS). For example, Hwang et al. [12] demonstrated that using an eye-gaze controlled device improved the quality of life of ALS patients and decreased caregiver burden, the latter of which may have resulted from more effective communication between patients and caregivers. In a telephone survey of 30 ALS patients [17], the reported median time of using an eye-gaze technology was 300 min per day. About 63% of the participants were defined as regular users, with a median use of 420 min per day for daily communication with relatives/caregivers as well as Internet surfing, emailing, and social networking. Caligari and colleagues [4] surveyed 35 adults in the late stages of ALS, who were regular users of eye-gaze technology communication devices, about their communication and quality of life. The study found that the devices were effective in reducing communication disabilities, improving quality of life, and producing high user satisfaction.

Research into children’s use of eye-gaze controlled computers for everyday activities has also been sparse. A longitudinal study of 10 participants aged 1–15 years-old with severe physical disabilities and complex communication needs demonstrated that all were able to use eye-gaze to control a computer and that the intervention (eye-gaze controlled computer and related services) successfully affected the predefined goals [13,18]. Qualitative studies by the same research group found that eye-gaze controlled computers increased children’s ability to express themselves and perform activities independently [19] and gave parents and teachers hope for a better future for the children [20].

To date, research into eye-gaze controlled computer for individuals with disabilities has been limited. Scholars have called for intervention studies [5] and studies that investigate users’ perspectives on usability after long-term use of the technology [21]. Additionally, studies involving more participants would increase the reliability and generalizability of the results, and that including both adults and children who use eye-gaze controlled computer would make it possible to examine similarities and differences between these groups. 

Accordingly, the current study aimed to identify prevalence and perceived usability in everyday life of eye-gaze controlled computer use among adults and children living in Sweden through utilization of a total population survey. This approach was chosen for the following reasons: (a) In Sweden, assistive technology, including eye-gaze controlled devices, deemed necessary for daily living is part of public healthcare and is free-of-charge [22]; (b) assistive technology centers are located in all 21county councils in Sweden, thus ensuring widespread access to the technology [23]; (c) assistive technology use is included as part of an individual’s health record, along with other characteristics (e.g., gender, age, type of disability); and (d) Sweden is home to one of the world’s leading developers of eye-tracking technology (Tobii), which began offering eye-gaze controlled products in 2006 (Tobii P10). 

The following research questions were asked: What are the characteristics of individuals who use the technology (e.g., age, gender, type of disability)? Are there differences between adults and children regarding disability, the use of eye-gaze controlled computer, and its perceived usability?

## 2. Materials and Methods 

### 2.1. Research Design

A total population survey [24] of individuals in Sweden who had received and were currently using eye-gaze controlled computer was conducted. The Regional Ethics Review Board in Linköping, Sweden (Dnr 2016/218-31), approved the study.

### 2.2. Participants

**Figure 1:**
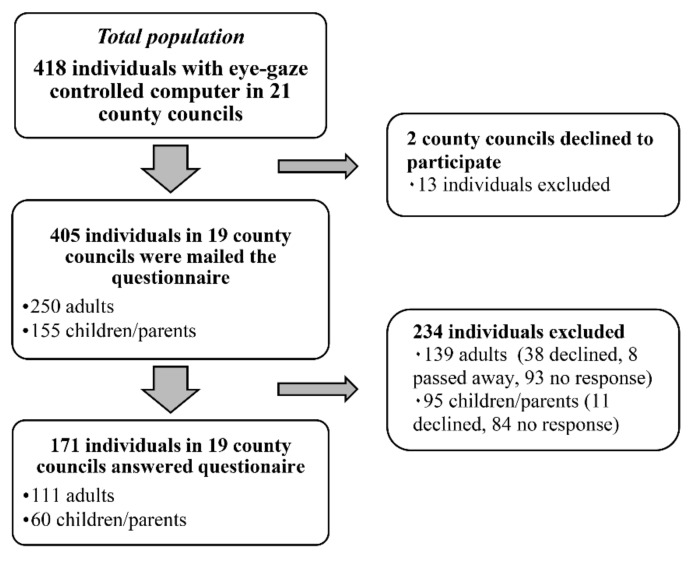
Flow chart of study population.

Inclusion criteria were inhabitants of all age groups (both adults and children <18 years old) in Sweden who, at the time of the study (2017), were using an eye-gaze controlled device provided by the county council’s assistive technology center. Officials at each center in all county councils (*n* = 21) in Sweden were contacted by email and requested to participate. The 21 centers reported in total 418 eye-gaze technology users. Of these 21 centers, two centers declined by email: one because there were no eye-gaze technology clients and the other (with 13 users) due to time constraints. The remaining 19 consented participation by email. In all, 405 users (250 adults and 155 children) were identified across the 19 centers. The centers were then asked to compile and develop coded lists documenting the users’ gender, age, type of device, date of delivery, and, if available, diagnosis, from the information available in their records. The research group provided each center with sealed envelopes, without addresses but with codes, based on the number of users reported by them. The envelopes included an information letter and a questionnaire on paper, with different envelopes for adult users (adult questionnaire) and parents of child users (child questionnaire). The questionnaire could be answered on paper or as a web-based questionnaire. A web link was, therefore, included in the information letter to provide an alternative of answering the web-based questionnaire using their eye-gaze controlled computer. The centers mailed the envelopes to the users in May 2017, with two mailed reminders within 8 weeks. Of the total 405 users, 171 consented to participate (adults, *n* = 111, children/parents, *n* = 60) by answering the questionnaire either by mail (*n* = 146) or web (*n* = 25). Parents responded on children’s behalf. Figure 1 provides a flow chart of the study population. Data were obtained from centers for 226 of 234 excluded individuals (non-respondents) since 8 adults had passed away.

### 2.3. Questionnaire

Two questionnaires were developed, namely one adult questionnaire and one child questionnaire, to investigate their perception of the usability of eye-gaze controlled computers (effectiveness, efficiency, satisfaction), and their use of eye-gaze controlled computer (activities, duration, and frequency of use). The questionnaire was comprised of three sections: (1) Personal Characteristics, (2) Use and Usability of Eye-gaze Controlled Computer in Everyday Life, and (3) Usability in Terms of Satisfaction with the Device and Related Services. Section 1 included 10 questions for adults and 15 questions for children on age, gender, school/work, diagnosis, communication modalities, and computer experience. Section 2 included seven questions on frequency and duration of use/day, type of activities, importance of activities, and usability in terms of efficiency (how effortful it is to use) and effectiveness (use in as many activities and as often as needed). Section 3 included 12 questions from the Quebec User Evaluation of Satisfaction with Assistive Technology (QUEST 2.0) scale [25].

Answers to Section 1 were noted on predefined categories (e.g., diagnosis) or were scored on ordinal scales (e.g., computer experience). In Section 2 all questions were scored on Likert scales. The questions on frequency and type of activity included 16 predefined activities, for which participants rated how often they performed each activity using the eye-gaze controlled computer on a 4-point scale ranging from 1 (daily) to 4 (seldom or never used) with the option to respond “not applicable” if needed. Likewise, the importance of the same 16 activities was scored on a 4-point scale from 1 (very important) to 4 (not important at all). The activities were playing/games, listening to music/radio, reading/looking in books, watching videos (e.g., YouTube), writing with letters, writing with symbols, talking with someone in the same room, using email, searching for information on the Internet, using social media, shopping on the Internet, using public services on the Internet, making phone calls, using environmental control, recording videos (children), doing homework/school tasks (children), doing studies/school work (adults), and performing work tasks (adults). An option to include a new activity was also given. These predefined activities were typical computer activities among adults [26] and children [27] in the general population as revealed by two national surveys, and among children with disabilities as demonstrated by an intervention study [13]. In the question about duration, participants rated the time spent using the eye-gaze controlled computer during a typical day in school/at work and during leisure, with six gradations from 1 (more than 8 h) to 6 (not used at all). The remaining three questions in Section 2 were; “How effortful is it usually for you/the child to use eye-gaze controlled computer?”, “I think I/the child use eye-gaze controlled computer in as much activities as needed”, and “I think I/the child use eye-gaze controlled computer as often as needed”.

For Section 3 (satisfaction), the Quebec User Evaluation of Satisfaction with Assistive Technology (QUEST 2.0) [25] was used, which is a 12-item measure assessing user satisfaction with assistive technology devices and services. It contains the question “How satisfied are you with…?” rated on a 5-point scale from 1 (Not satisfied at all) to 5 (Very satisfied) for eight device items (e.g., easy to use) and four service items (e.g., follow-up services).

### 2.4. Procedure

Before data collection, the questionnaire was pilot-tested on one adult user and a parent of a child user, which resulted in minor changes in language and, in the children’s version, specification, of different kinds of assistants in school [24]. Think-aloud interviewing was used [28] via telephone after they had completed the questionnaire.

### 2.5. Data Analysis 

In Section 1, the reported diagnoses were categorized into eight diagnostic groups (see Table 1) for respondents and non-respondents. Descriptive statistics were calculated, with mean and standard deviations describing the participants’ age and years of access to an eye-gaze controlled computer. Frequencies were used to describe other data on participants’ characteristics (e.g., different types of communication modalities, degree of assistance). For Section 2, frequencies were calculated for the use of the eye-gaze controlled computer (e.g., frequency of use). Frequencies of use for each specified activity were then categorized into daily (every day) or weekly use (once per week and several times per week) for each participant. Activity repertoire was calculated for each participant by counting all activities performed using the eye-gaze controlled computer on either a daily or weekly basis, including both predefined activities and new activities if added by participants. The use for Internet-related activities was dichotomized as “yes” (daily or weekly use) and “no” on an individual level, with “yes” meaning use in one or more of the activities (e.g., social media; see questionnaire). Cronbach’s alpha calculated for Section 2 (Use and Usability of Eye-Gaze Controlled Computer) of the questionnaires, yielded the following values: α = 0.92 for adults and α = 0.91 for parents. In QUEST (Section 3), following Demers et al. [25], each item scored using the 5-point satisfaction scale was categorized as either dissatisfied (1 = not at all, 2 = not very, and 3 = more or less) or satisfied (4 = quite satisfied and 5 = very satisfied), and presented with frequencies. 

For the response analyses, the code lists from the assistive technology centers were used, and chi-squared tests (diagnosis group, gender) and independent *t*-tests (age, years using eye-gaze controlled computer) were computed to examine any differences between the respondents and non-respondents.

To determine the differences across variables between adults and children (<18 years old), chi-squared tests (e.g., control methods, daily or weekly usage), Mann–Whitney U–tests (e.g., duration of use, degree of assistance), and independent *t*-tests (e.g., activity repertoire, years using eye-gaze controlled computer) were used. All percentage calculations were based on the number of participants answering each question. Missing values of each item ranged between 0 and 7 for children and 0 and 12 for adults.

## 3. Results

The assistive technology centers identified 418 individuals who currently had access to an eye-gaze controlled computer, giving a prevalence of 0.042% in the Swedish population. The response rate was 41%. The analysis showed no significant differences between respondents (*n* = 171) and non-respondents (*n* = 226) regarding age (*t*(394) = 0.736, *p* = 0.462), gender (χ2 (1, *n* = 397) = 0.372, *p* = 0.542), years of using the prescribed eye-gaze controlled device (*t*(391) = 1.260, *p* = 0.208), or the proportion of persons with various diagnoses (χ2 (6, *n* = 336) = 9.524, *p* = 0.146). The diagnosis was specified for only 168 of the 226 non-respondents because not all centers had access to each individuals’ diagnosis.

The results are organized in the three sections, namely (1) Participants Characteristics, (2) Use and Usability in Everyday life, and (3) Satisfaction with the Device and Related Services.

### 3.1. Section 1: Participant Characteristics

Participant characteristics are shown in Table 1. The participants’ ages ranged between 4 and 81 years, with the majority being of school or working age (M = 30.9, SD = 19.7). Of the 171 participants, 84 (49%) were females. Among adults, 23 (21%) reported that they worked; among children, 45 (75%) attended a special school, while 15 (25%) attended a mainstream school. All participants were dependent on assistance and had access to an eye-gaze controlled computer for 2 years (SD = 1.7, range: 0–8 years). Cerebral palsy (*n* = 78, 45.6%) was the most common diagnosis across both adult and child participants, with more children (*n* = 46, 76.7%) than adults (*n* = 32, 28.8%) reporting this diagnosis. The next most common diagnosis was amyotrophic lateral sclerosis (ALS) (*n* = 29, 26.1%) for adults and Rett syndrome (*n* = 10, 16.7%) for children. Concomitant impairments were present for 20% of participants, of which the most frequent were visual impairments, reported by 12% with either cerebral palsy or brain injury. Regarding computer experience, 102 of 168 (61%) of the participants perceived themselves as having either adequate or considerable experience, while 19 (11%) reported no experience at all. Among children, parents rated their own computer experience as significantly higher than the adult users (U (*n* = 168) = 2613.00, z = –2.13, *p* = 0.033).

#### 3.1.1. Communication

Regarding communication modalities, 109 (71%) participants did not communicate with others using speech, 117 (73%) used the eye-gaze controlled device, and 94 (60%) used communication boards to communicate. Analysis showed that adult users communicated using speech to a higher extent than children (χ2 (1, *n* = 153) = 12.68, *p* < 0.001), while the children communicated using communication boards to a higher extent than adults (χ2 (1, *n* = 156) = 18.48, *p* < 0.001). Among the participants, 99 (58%) were totally dependent on assistance from another person for face-to-face communication, and 30 (18%) reported considerable need of assistance, whereas 19 (11%) reported some need of assistance and 22 (13%) reported no need of assistance at all. Children were rated as more dependent on assistance to communicate with others than adults (*U* (*n* = 170) = 2425.00, z = –3.20, *p* = 0.001).

#### 3.1.2. Eye-Gaze Technology Devices

Of 171 participants, 165 (96%) had access only to one eye-gaze technology device, while six participants (4%) had access to two devices. The accessibility duration of eye-gaze controlled computer ranged up to 8 years, with a mean duration of 2 years (SD = 1.7). The number of systems and duration of eye-gaze controlled computer access did not differ between adults and children (*p* > 0.05). Among participants, 160 (94%) had different Tobii eye-gaze controlled devices (C-series, I-series, P10, PCeye Go, PCeye mini). The Tobii I-series product were most common, used by 95 participants (58%). Other companies’ eye-gaze controlled computer products (Rolltalk Intelligaze, Powerbox 7, or Grid Pad Eye) were used by 11 (6%) of the participants. The proportions of participants using different products did not differ between adults and children (*p* > 0.05). If the participants perceived difficulties with the eye-gaze controlled computer, 157 of 169 participants (93%) reported having someone near who could help them. More adult users (*n* = 106, 97%) reported having someone who could help them than did the parents (*n* = 51, 85%) (χ2 (1, *n* = 169) = 8.80, *p* = 0.003).

#### 3.1.3. Control Methods other than Eye-Gaze

Of 168 participants, 125 (74%) had no other way to control a computer than through eye-gaze, while 23 (14%) could also use their hands. Only a few used other control methods (e.g., head movements, voice recognition). The analysis revealed no differences between adults and children in the proportion of participants using different methods (*p* > 0.05). 

### 3.2. Section 2: Use and Usability in Everyday Life

#### 3.2.1. Frequency and Duration of Use

Of the 171 participants, 164 (96%) reported a frequency of eye-gaze controlled computer use of at least every week or more often; 108 (63%) had daily usage, and 56 (33%) had weekly usage, while seven (4%) reported seldom or no use at all. The proportions of participants with daily use and weekly use showed no differences between adults and children (*p* > 0.05).

Of the 165 participants, 149 (90%) participants reported using the eye-gaze controlled computer during leisure times, with significantly longer durations among adults than children (*U* (*n* = 165) = 2026.50, z = –3.71, *p* < 0.001). As shown in Table 2, 39 (68%) of the children had leisure use of up to 2 h a day, while 49 (45%) of the adults reported longer durations, such as more than 2 h. A few participants, including three children (5%) and 17 adults (16%), had leisure use of more than 8 h per day.

For child participants, the duration of use was higher in school than during leisure; 15 (26%) used the eye-gaze controlled computer daily at school for more than 2 h, while 10 (18%) used it for the same duration during leisure. Among children, 48 (83%) used it during schoolwork, and 33 (57%) used it generally for up to 2 h during a school day. Among adults, 54 (54%) reported using the eye-gaze controlled computer during work; of these participants, 15 (15%) used it for 4 h or more. Four adults (3%) and two child participants (3%), used it for more than 8 h per day at work or in school.

#### 3.2.2. Activities

The activity repertoire for each person ranged up to 12 activities (*n* = 171, M = 4.8, SD = 3.0, range: 0–12). The activity repertoire showed no differences between adults and children (*p* > 0.05). The most common activity using eye-gaze controlled computer (every week or more often) across all participants was to talk (111 of 169, 66%). Some differences were found in the two most common activities between children and adults. While most adults used the eye-gaze controlled computer to write (73 of 111, 66%) and talk (68 of 109, 62%), the most common activities among the 60 children were playing/games (*n* = 47, 78%) followed by talking (*n* = 43, 72%).

Use of the eye-gaze controlled computer for Internet-related activities (email, searching for information, social media, shopping, public services) was reported by 49% (54 of 110) of adults and 13% (8 of 60) of children. Among adults, all five predefined Internet-related activities were reported, while two of them (searching for information, social media) were reported among children. The two activities rated by most participants as very important to perform using the eye-gaze controlled computer were to talk (84 of 162, 52%) and write with letters and/or symbols (82 of 164, 50%), which were also the most important among adult users. Among children, 57% (33 of 58) of parents rated talking as most important, followed by play and games (25 of 58, 43%).

#### 3.2.3. Efficiency 

Concerning the efficiency of using an eye-gaze controlled computer, 98 of 168 (58%) participants recorded the use as either not effortful or only to some extent. The use was exhausting for eight (5%), while 62 (37%) found it to be quite effortful (Table 2). There were no significant differences between adults and children regarding the ratings of effort (*p* > 0.05).

#### 3.2.4. Effectiveness

Table 2 also displays the effectiveness, as indicated by the extent to which the use of the eye-gaze controlled computer corresponded with the perceived needs. The analyses revealed that adults use the eye-gaze controlled computer to a greater extent in all activities as needed than children (*U* (*n* = 159) = 2147.00, z = −2.89, *p* = 0.004). Among children, 69% (40 of 58) use the eye-gaze controlled computer only to some extent or not at all, due to their activity requirements. Likewise, adults use the technology as often as needed to a higher extent than children (*U* (*n* = 159) = 1840.50, z = −3.91, *p* < 0.001). Among children, 63% (35 of 56) used it only to some extent or not at all, due to the frequency of use.

### 3.3. Section 3: Satisfaction with the Device and Related Services

Overall, the results revealed that the 161 participants (104 adults, 57 children/parents) who answered the QUEST 2.0 were quite satisfied with the eye-gaze controlled computer (total scale; M = 3.7, SD = 0.8), with high satisfaction ratings for the device (M = 3.8, SD = 0.8) and somewhat lower satisfaction with the county council’s service delivery (M = 3.6, SD = 1.1). The analysis also showed that device satisfaction did not differ between the groups (*p* > 0.05), while adults were more satisfied with the services than parents (U (*n* = 161) = 2282.00, z = −2.42, *p* = 0.015). The same differences were found for the total scale (U (*n* = 161) = 2348.50, *z* = −2.18, *p* = 0.029) (for details, see Table 2). The items with the most satisfied participants were dimensions (132 of 162, 81%), safety (122 of 157, 78%), and durability (122 of 158, 77%), whereas the items with the most dissatisfied users were follow-up services (78 of 164, 48%), weight (80 of 161, 50%), and comfort (62 of 159, 39%).

## 4. Discussion

This study investigated the prevalence and usability of eye-gaze controlled computers from the users’ perspective within the whole population of children and adults who received it as an assistive technology in Sweden. The results of the current study demonstrate that an eye-gaze controlled computer is an important device for participants’ interaction and communication with others, and that face-to-face communication was the most commonly used communication method across the participants. Writing and Internet-related activities were also common and considered as activities having social and communication purposes [29]. Thus, an encouraging finding was that eye-gaze controlled computer is used to fulfill everyday communication needs among school- and working-age individuals with severe physical disabilities and complex communication needs. Communicating to express one’s opinions is a basic human right according to Article 19 of the Universal Declaration of Human Rights [30], and access to and use of augmentative and alternative communication devices (AAC) by individuals with complex communication needs is crucial for participating in work, school, and community life [31]. Thus, an eye-gaze controlled computer seems to be a device used widely in real life by this population, facilitating their participation in both the digital and social worlds.

In Sweden, a country with about 10 million inhabitants, the assistive technology centers identified 418 individuals who currently had access to an eye-gaze controlled computer, providing a prevalence of 0.042%. The results demonstrate that an eye-gaze controlled computer was used by individuals of all ages and equally by males and females. Most of the participants were in an active phase of their lives, either of school-aged or working age, with a mean age of 30.9 years (SD = 19.7). All were dependent on assistance and had complex communication needs; only a few used a method other than eye-gaze to control their device. Thus, considering the groups’ difficulties, no indications of over-prescription were found. Rather, the results indicate that people with less profound disabilities, for whom the eye-gaze technology could be an alternative to control a computer with higher efficacy, do not receive the device as an assistive technology [21]. A few survey studies in different countries have indicated an excessively low access rate of eye-gaze technology because not all individuals with severe disabilities in need of such devices are afforded access to it. For example, a survey study of individuals with ALS in Germany found a rejection rate of 48% by health insurance institutions concerning communication devices such as eye-gaze controlled computer [32]. A survey in the Netherlands on families with a child with Rett syndrome [33] and a study of speech-language therapists in Sweden who support individuals with Rett syndrome [34] show that not all of these individuals have an opportunity to try eye-gaze technology to see if they may benefit from its use.

When comparing the results of child and adult users of eye-gaze controlled computer, differences in use between them must be understood in the light of age, diagnosis, and onset of disability as well as contextual circumstances. For example, younger age, cognitive impairments, or inadequate support might reduce the number of applications an individual can use and thereby reduce their motivation to use the computer. The results demonstrate that the distribution of diagnoses was uneven between the adults and children. About 93% of the children had cerebral palsy or Rett syndrome, which is diagnoses indicating variations in cognitive abilities, as well as a loss of motor control [35,36]. These diagnoses debut in early childhood, while the diagnoses of about 40% of adult users (e.g., ALS, stroke, multiple sclerosis) typically occur in adulthood [37], which implies that most of the adults in the current study had already learned how to read, write, communicate, and control a computer, before they received their device. For these individuals, eye-gaze technology made it possible to continue to perform these previously learned skills and activities. For most of the children, however, an eye-gaze controlled computer might have been necessary for learning basic skills, such as reading and communicating. Although the distributions of diagnoses were different, cerebral palsy was the most common diagnosis in both children (76.7%) and adults (28.8%). The finding that cerebral palsy was the most common diagnosis among adults was interesting because existing research on adults almost exclusively concerns individuals diagnosed with ALS [4,12,17], which was the second most common diagnosis (26.1%) among adults in the current study. Based on this result, a recommendation for future research on eye-gaze technology use in adults is to focus on persons with cerebral palsy, as their needs and challenges might be different from those of people diagnosed with a progressive disease as adults.

Findings show that overall, adults used eye-gaze controlled computer more often and for longer durations than the child participants during leisure time and used the device to a higher extent for the activities that they wanted to perform (adults, 59% vs. children, 31%) and needed most (adults, 65% vs. children, 38%). High users (use of more than 8 h per day) included 16% of adults and 5% of children. Moreover, even though many children (72%–74%) used an eye-gaze controlled computer for activities they rated as most important (57% to talk; 43% for play activities and games) and were regular users, a majority of parents either did not agree or agreed only to some extent that children’s usage matched the needed number of activities (69%) and frequency of use (63%). From the parents’ perspective, the results indicate that eye-gaze technology is not as effective for a majority of children. Issues of accessibility might explain the lower computer use/day among children than adults. Nearly all participants had access to only one device that is difficult to set up [21], which might have influenced the usage in the two settings. For example, children reported a higher use in school than at home that, in turn, might be a result of only having access to an eye-gaze controlled computer in school. Adults had a higher use per day during leisure time than children, which might be because a majority of adult users were on sick leave because of which they did not have the same need for multiple devices. The results also reveal that both adults and children were quite satisfied with the eye-gaze controlled computer, although the parents were less satisfied with the associated services than adult users. Fifty eight percent of participants reported the eye-gaze controlled computer as an efficient assistive technology for daily life use. Still, 42% reported the device to be totally or quite effortful to use. Factors affecting the efficiency of using the eye-gaze controlled computer is not investigated in this study and is a recommendation for future research. Earlier studies have reported that eye strain is a common problem for those using eye-gaze technology [17]. Service providers should, therefore, consider whether and how the adaptations of the device applications can reduce the individual’s perception of effort during use. Based on eye-tracking research in augmentative and alternative communication applications, Light et al. [38] argued that even small changes in visual arrangements in grids could enhance the visual search and eye-motor behavior of individuals with developmental or acquired disabilities when using the applications. Results show that although both the adult and child participants in this study regularly used their eye-gaze controlled computer, adults reported greater frequency of use for activities they wanted and needed to perform, and said they were more satisfied with services compared to the parent respondents. Considering the identified differences between adults and children, these results, in turn, indicate that service providers need to prioritize children as eye-gaze technology users and learn more about their needs and specific circumstances to develop more effective services for children and their parents.

### Limitations

As a total survey, this study reached 41% of the target population of people who had received and were currently using an eye-gaze controlled computer in Sweden. The study used a postal/web survey for data collection and it is likely that the target groups’ complex communication needs and severe disabilities reduced the response rate [39], particularly among adult users who might have to answer for themselves using the eye-gaze controlled computer. Conducting a survey with personal interviews often raises the response rate [39], but the complex communication needs in this population ruled this out as an option. Non-response analysis found no differences between respondents and non-respondents in age, gender, or diagnosis. The results might be generalizable, although with caution.

Another limitation is that the child-participant questions were answered by their parents, who often report more accurately on areas they can easily observe, such as activities in the home setting rather than activities in school [40]. If parents did not consult with their children, the information about school use might be flawed, especially considering that eye-gaze control computer use was reported to be higher in school than in the home setting.

## 5. Conclusions

The results demonstrate the usability of eye-gaze controlled computers both at school and work and during leisure for people with severe physical disabilities and complex communication needs of all ages. The device was used primarily for communication but also enabled partaking in a wide range of Internet activities, indicating that the device is important for participation at both the individual and societal level. Overall, adults perceived higher usability of eye-gaze controlled computers than children. They reported higher and more effective use and were more satisfied with services than children, indicating that applications and services, particularly for children, can be improved.

## Figures and Tables

**Table 1:** Participants’ and non-respondents’ characteristics.

Characteristics	Participants						Non-Respondents	
	All		Adults		Children			
	(*n* = 171)		(*n* = 111)		(*n* = 60)		(*n* = 226)	
	M	SD	M	SD	M	SD	M	SD
Age	30.9	19.7	40.99	17.5	12.5	4.2	29.4	20.3
	*n*	%	*n*	%	*n*	%	*n*	%
Gender								
Female/Male	84/87	49/51	56/55	50.5/49.5	28/32	47/53	118/108	52/48
Personal assistant	145	86	93	86	52	87		
Diagnosis								
Cerebral palsy	78	45.6	32	28.8	46	76.7	74	44.0
Amyotrophic lateral sclerosis	29	17.0	29	26.1			27	16.1
Rett syndrome	23	13.5	13	11.7	10	16.7	19	11.3
Stroke, brain injury	13	7.6	12	10.8	1	1.7	7	4.2
Muscular dystrophy	11	6.4	9	8.1	2	3.3	13	7.7
Multiple sclerosis	7	4.1	7	6.3			7	4.2
Spinal cord injury	3	1.8	3	2.7				
Other ^a^	7	4.1	6	5.4	1	1.7	21	12.5
Work	23	13	23	21				
School ^b^								
Special school	45	26			45	75		
Mainstream school	15	9			15	25		
	M	SD	M	SD	M	SD	M	SD
Eye-gaze controlled computer access in years	2.0	1.7	2.0	1.8	2.1	1.6	1.8	1.7

Note. Non-respondents with diagnosis, n = 168. ^a^ Other: e.g., Mitochondrial disease, thromboembolic disease, Huntington disease, neurological problem, severe intellectual disability, Ehlers–Danlos syndrome, unspecified physical disability; ^b^ School: Compulsory school and Upper Secondary school.

**Table 2:** Responses on some of the use and usability questions in the questionnaire.

Variables	All		Adults		Children		Group Comparison
	*n*	%	*n*	%	*n*	*%*	*p*
USAGE							
Duration leisure	(*n* = 165)		(*n* = 108)		(*n* = 57)		<0.001 (children
Not at all	16	9.7	8	7	8	14	longer
Up to 2 h	90	54.6	51	47	39	68	duration)
>2 h <4 h	26	15.8	21	19	5	9	
4h or more	33	20	28	26	5	9	
Duration work/school	(*n* = 157)		(*n* = 99)		(*n* = 58)		NA
Not at all	55	35	45	46	10	17	
Up to 2 h	60	38	27	27	33	57	
>2 h < 4 h	20	13	12	12	8	14	
4 h or more	22	14	15	15	7	12	
EFFICIENCY							
“How effortful is it usually for you/the child to use EGCC?”^§^	(*n* = 168)		(*n* = 108)		(*n* = 60)		ns
Not at all/some	98	58.4	67	62.0	31	51.7	
Quite much	62	36.9	37	34.3	25	41.7	
Totally	8	4.8	4	3.7	4	6.7	
EFFECTIVENESS							
“I think I/the child use EGCC in as much activities as needed”	(*n* = 159)		(*n* = 101)		(*n* = 58)		0.004 (adults higher extent)
Agree totally/ to a large extent	78	49	60	59	18	31	
Agree to some extent	46	29	20	18	26	45	
Not at all	35	22	21	19	14	24	
“I think I/the child use EGCC as often as needed”	(*n* = 159)		(*n* = 103)		(*n* = 56)		<0.001 (adults, higher extent)
Agree totally/ to a large extent	88	55	67	65	21	38	
Agree to some extent	40	25	20	19	20	36	
Not at all	31	20	16	16	15	27	
	M	SD	M	SD	M	SD	*p*
SATISFACTION	(*n* = 159–161)		(*n* = 103–104)		(*n* = 56–57)		
QUEST Total scale	3.75	0.76	3.83	0.77	3.72	0.63	0.029
QUEST Device subscale	3.80	0.76	3.84	0.83	3.40	1.01	ns
QUEST Service subscale	3.65	1.07	3.78	1.08	3.60	0.72	0.015

Note. NA: not applicable; ns: not significant; ^§^ EGCC: Eye-gaze controlled computer.
